# Plastid: nucleotide-resolution analysis of next-generation sequencing and genomics data

**DOI:** 10.1186/s12864-016-3278-x

**Published:** 2016-11-22

**Authors:** Joshua G. Dunn, Jonathan S. Weissman

**Affiliations:** 1 California Institute of Quantitative Biosciences, San Francisco, USA; 2 Department of Cellular and Molecular Pharmacology, University of California San Francisco, San Francisco, CA USA; 3 Howard Hughes Medical Institute, University of California San Francisco, San Francisco, CA USA; 4 Center for RNA Systems Biology, Berkeley, CA USA

**Keywords:** Sequencing, Genomics, Bioinformatics, Python, Ribosome profiling

## Abstract

**Background:**

Next-generation sequencing (NGS) informs many biological questions with unprecedented depth and nucleotide resolution. These assays have created a need for analytical tools that enable users to manipulate data nucleotide-by-nucleotide robustly and easily. Furthermore, because many NGS assays encode information jointly within multiple properties of read alignments ― for example, in ribosome profiling, the locations of ribosomes are jointly encoded in alignment coordinates and length ― analytical tools are often required to extract the biological meaning from the alignments before analysis. Many assay-specific pipelines exist for this purpose, but there remains a need for user-friendly, generalized, nucleotide-resolution tools that are not limited to specific experimental regimes or analytical workflows.

**Results:**

Plastid is a Python library designed specifically for nucleotide-resolution analysis of genomics and NGS data. As such, Plastid is designed to extract assay-specific information from read alignments while retaining generality and extensibility to novel NGS assays. Plastid represents NGS and other biological data as arrays of values associated with genomic or transcriptomic positions, and contains configurable tools to convert data from a variety of sources to such arrays.

Plastid also includes numerous tools to manipulate even discontinuous genomic features, such as spliced transcripts, with nucleotide precision. Plastid automatically handles conversion between genomic and feature-centric coordinates, accounting for splicing and strand, freeing users of burdensome accounting. Finally, Plastid’s data models use consistent and familiar biological idioms, enabling even beginners to develop sophisticated analytical workflows with minimal effort.

**Conclusions:**

Plastid is a versatile toolkit that has been used to analyze data from multiple NGS assays, including RNA-seq, ribosome profiling, and DMS-seq. It forms the genomic engine of our ORF annotation tool, ORF-RATER, and is readily adapted to novel NGS assays. Examples, tutorials, and extensive documentation can be found at https://plastid.readthedocs.io.

## Background

Next generation sequencing (NGS) has transformed biology. Beyond enabling the rapid sequencing of genomes, increasingly sophisticated NGS assays have empowered biologists to probe a wide array of biological processes with unprecedented precision and depth, provided that the desired information can be encoded within a nucleic acid sequence. Many NGS assays encode nucleotide-resolution information within multiple properties of sequencing reads ― such as their alignment coordinates, lengths, or sites at which they mismatch a reference sequence ― and thus require analytical tools that decode biological data from such properties. One such assay is ribosome profiling, in which the positions of the ribosomal P-sites are jointly encoded by the lengths and positions of aligned sequencing reads [1, 2]. Another example is bisulfite sequencing, in which the methylation status of cytosine residues is encoded in the genomic locations of C-to-T transitions within read alignments [3, 4].

Because decoding biological information from read alignments is not trivial, a wide array of software has been developed for this purpose. At one extreme are tools dedicated to specific, predefined analysis of data from a single assay, such as riboSeqR [5], RiboTools [6], and RiboGalaxy [7] for ribosome profiling, or PROTEOFORMER [8], ORFscore [9], or ORF-RATER [10] for de novo protein discovery and ORF annotation. Tools like these are user-friendly, but, as a consequence of their design, are difficult to adapt to novel purposes. At the other extreme are low-level, generalized tools, like SAMtools [11] and BEDtools [12], that are not designed for or limited to any specific assay or experimental setup. These tools are extremely powerful, but using them requires substantial expertise in programming and awareness of seemingly esoteric file formats. Between these extremes lie a number of user-friendly and general-purpose toolkits, such as HTSeq [13], Metaseq [14], bx-python [15], and Bioconductor [16]. But these, in their present forms, are limited in their abilities to decode information from raw read alignments, to manipulate (or, in some cases, even to represent) discontinuous genomic features such as multi-exon transcripts, or to perform nucleotide-resolution analysis. The situation is further complicated by the fact that many file formats have been invented to describe only a handful of data types in genomics (Table 1), and that even synonymous file types can be treated inconsistently within toolkits.

**Table 1:** File formats used in genomics

Data type	Format	Implementation
Feature annotations (e.g. genes, transcripts, exons, origins of replication)	BED, extended BED*	Plastid
	BigBed	Plastid + kentUtils [46]
	GTF2*	Plastid
	GFF3*	Plastid
	PSL*	Plastid
Read alignments	bowtie	Plastid
	BAM	Plastid + Pysam [27]
Reduced count data	bedGraph	Plastid
	BigWig	Plastid + kentUtils [46]
	wiggle (fixedStep)	Plastid
	wiggle (variableStep)	Plastid
Sequence	FASTA	via Biopython [20]
	twobit	via twobitreader [21]

For each category of genomics data, many file formats exist. Plastid includes readers for each format that standardize the representation of data for each type, so that the meaning of each data type is separated from its format on disk. *tabix compression for these formats is supported via Pysam [27]

**Fig. 1:**
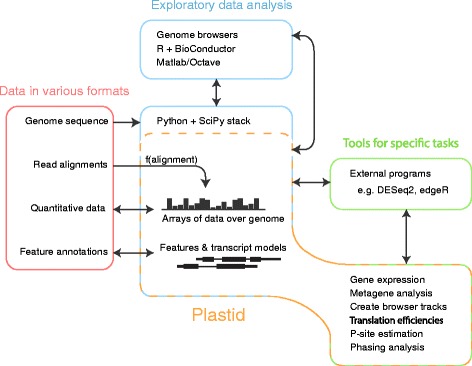
Uses of Plastid in analysis workflows. Plastid (*yellow box*) contains tools for both exploratory data analysis (*blue*, *center*) and command-line scripts for specific tasks (*green, right*). Plastid standardizes representation of data across the variety of file formats used to represent genomics data (*left*). Quantitative data are represented as arrays of data over the genome. Read alignments may be transformed into arrays using a mapping function appropriate to a given assay. Transcripts are represented as chains of segments that automatically account for their discontinuities during analysis. Plastid integrates directly with the SciPy stack (blue, center). For exploratory analysis in other environments (blue, above) or further processing in external programs (*right*, *green*), Plastid imports and exports data in standardized formats

**Fig. 2:**
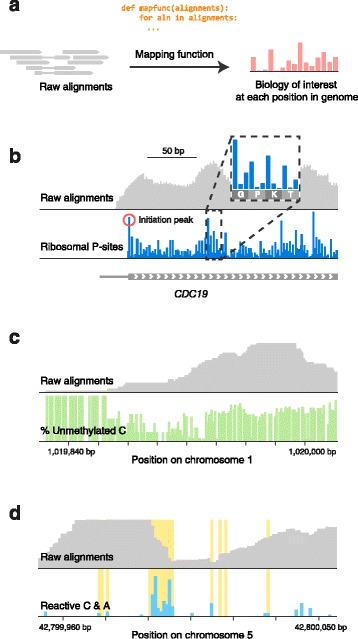
Mapping functions extract biological data from read alignments. **a**. Mapping functions use various properties of a read alignment to determine the genomic position(s) at which it should be counted. **b**. Mapping functions for ribosome profiling use alignment coordinates and lengths to estimate ribosome positions, revealing features of translation, like a peak of density at the start codon (*red circle*) and three-nucleotide periodicity of ribosomal translocation (inset). **c**. For bisulfite sequencing, the fraction of C-to-T transitions at each cytosine are mapped, revealing a CpG island. **d**. A mapping function for DMS-seq differentiates structured from unstructured regions of a selenocysteine insertion element in the 3′ UTR of human *SEPP1.* DMS reactivity (blue bars) matches A and C residues predicted to be unstructured (*yellow*)

Here we introduce Plastid, a Python library for nucleotide-resolution analysis of genomics data. Plastid is designed to retain the user-friendliness of pipeline tools designed for specific NGS assays, like RiboGalaxy, without sacrificing the generality and power of low-level tools, like BEDtools. Given its goals, Plastid’s design differs substantially from existing packages (Fig. 1):

First, Plastid’s internal analysis pipeline introduces a stage in which *mapping functions* extract the relevant biological information from various properties of raw read alignments. Biological data are then exposed to users as vectors of information ― such as the number of ribosomal P-sites or C-to-T mismatches found at each nucleotide position ― rather than lists of read alignments or vectors of raw coverage. Because mapping functions can perform arbitrary transformations on properties of read alignments, they add substantial flexibility to Plastid’s design: a mapping function suited to a given NGS assay tailors Plastid’s tools to that assay (Fig. 2). Uniquely, Plastid’s mapping functions are configurable and replaceable rather than hard-coded. Thus, Plastid has been used to analyze data from numerous types of experiments, including ribosome profiling, RNA-seq, DMS-seq, and bisulfite sequencing, and can be used for other assays (e.g. ChIP-seq, CAGE-seq, pseudouridine profiling) simply by choosing appropriate parameters for existing mapping functions, or by implementing new ones.

**Fig. 3:**
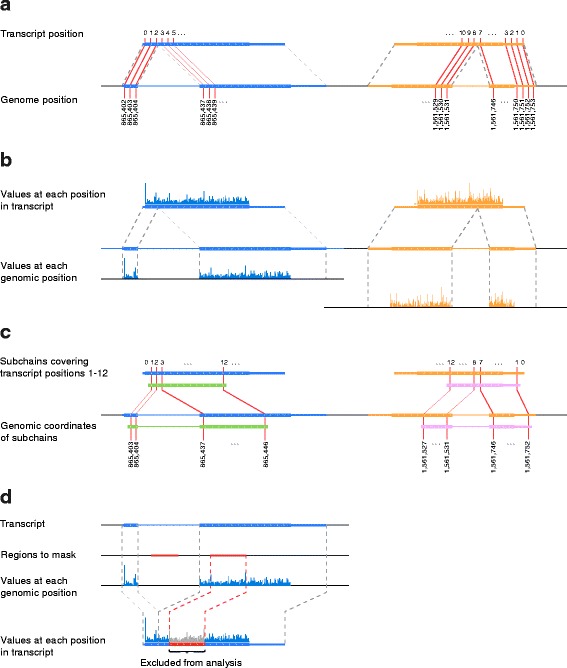
SegmentChains automate many common tasks. **a**. SegmentChain and Transcript objects automatically convert coordinates between genomic and transcript-relative spaces. **b**. SegmentChains and Transcripts can therefore convert read alignments or quantitative data aligned to the genome to arrays of values at each position in the chain. **c**. Subsections (*green, pink*) of chains can be fetched using start and end points relative to the parental chains. SegmentChains automatically generate the corresponding genomic coordinates. **d**. Regions of a chain can be masked from computations without altering the chain coordinates

Second, Plastid introduces a novel data model, the *SegmentChain,* to describe multi-exon transcripts and other discontinuous features. SegmentChains are aware of their own discontinuity and use this awareness to encapsulate many nucleotide-wise operations that are absent from other toolkits, such as conversion of coordinates or vectorized data between genomic and transcript-centric spaces. SegmentChains automatically account for splicing and complementing, and thus reduce user error during many tasks common in position-wise analysis (Fig. 3).

Third, Plastid provides consistent representations and behaviors for the various categories of genomic data, regardless of underlying file formats. Plastid’s tools thus enable users to focus on biological questions rather than data representation (Fig. 1, Table 1).

**Fig. 4:**
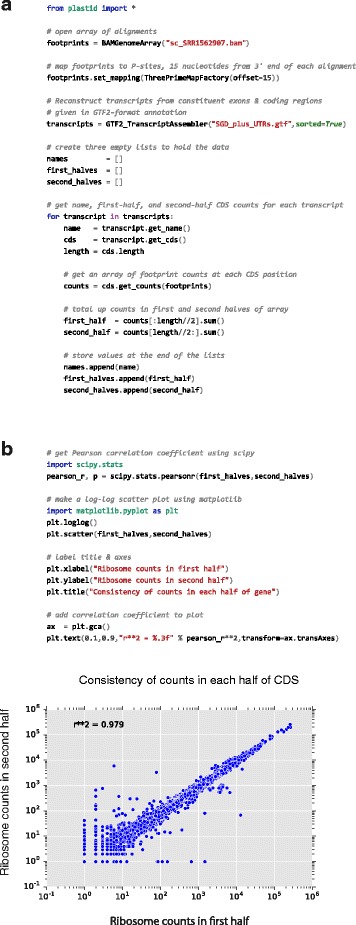
Plastid streamlines analysis. **a**. The quality of a ribosome profiling dataset may be assayed by comparing the numbers of read counts in the first versus second half of each coding region. Plastid makes it possible to implement such analyses with few lines of easily readable code. **b**. Plastid readily integrates with the tools in the SciPy stack. Here, first- and second-half counts from (**a**) are plotted against each other using matplotlib, and a Pearson correlation coefficient calculated using SciPy

Finally, Plastid’s intended audience includes bench scientists and novices as well as seasoned bioinformaticians. For this reason, Plastid defines a minimal sets of data structures that, when possible, have human-readable names and are modeled on biological objects — such as spliced transcripts — rather than on more abstract notions. Users can thus leverage their biological knowledge when writing or reading code (Fig. 4).

In addition to tools for nucleotide-resolution exploratory data analysis (EDA), Plastid includes command-line scripts that automate analysis workflows used for a number of common NGS assays, such as RNA-seq and ribosome profiling. Unlike similar implementations found in other toolkits, Plastid’s scripts leverage mapping functions, so that even common tasks, such as export of browser tracks for visualization of data, may be tailored to a specific biological question: for example, depending on the mapping function in use, Plastid’s *make_wiggle* script can export a browser track of mapped ribosomal P -sites, modified nucleotides, or any other data encoded within the read alignments, instead of simple read coverage. Like the rest of Plastid’s tools, these scripts can be generalized to novel assays with the implementation of new mapping functions.

Together, Plastid’s features enable novice and advanced users to develop analytical workflows that are both concrete and sophisticated, using familiar idioms and few lines of code. To support our users’ efforts, we offer extensive documentation, step-by-step walkthroughs of various analysis tasks, and a demo dataset for those walkthroughs at https://plastid.readthedocs.io.

## Implementation

### Representation of quantitative data

Many NGS assays encode nucleotide resolution data, and effectively associate a quantitative value with each genomic or transcriptomic position. A natural representation for such data is a vector or array of values, each position in the array corresponding to a nucleotide within a region of interest.

Plastid adopts this representation and represents quantitative data associated with genomic positions – such as the number of sequencing reads aligned to a given position, a phylogenetic conservation score, or local G/C content – using objects called *GenomeArrays*. Within GenomeArrays, data are indexed by chromosome, nucleotide position, and strand, and may be accessed via a Python dictionary-like interface using a SegmentChain as a key. GenomeArrays return data in an array format (NumPy array; [17]) whose positions correspond to nucleotide positions in the given regions of interest. The use of NumPy arrays enables the data to be used by the vast library of scientific tools compatible with the SciPy (Scientific Python) stack [18], and thus creates a useful bridge between genomics data and existing scientific infrastructure in Python.

Plastid includes implementations of GenomeArrays tailored to a number of file formats, including bedGraph, BigWig, and fixed-step or variable-step wiggle (Table 1). With the aid of mapping functions, GenomeArrays can also import read alignments in BAM or bowtie formats, performing transformations at runtime (for BAM files), or upon import (for bowtie files).

### Transformations of read alignments

Plastid’s GenomeArrays are designed to perform transformations on read alignments transparently during analysis, in order to extract the relevant biology — such as a nucleotide modification or ribosomal P-site — from whichever read properties encode them. These transformations are implemented in configurable mapping functions that determine the genomic position(s) at which the biology encoded in each alignment should ultimately be counted (Fig. 2a). Concretely, mapping functions are modular components of GenomeArrays take as input a query region of the genome and a set of read alignments, and return as output an array of transformed data covering each nucleotide position in the query region. Because mapping functions can exploit any property of a read alignment — for example, its length or sequence — in addition to its aligned positions, they provide a high level of flexibility and enable reuse of Plastid’s central tools with data from a large variety of NGS assays.

Mapping functions are particularly important to assays in which secondary properties of read alignments encode the biology of interest: for example, mapping functions for ribosome profiling assign counts to ribosomal P-sites, which occur at fixed offsets from the 5′ ends of read alignments, potentially varying as a function of read length [1]. P-site mapping reveals phenomena that are obscured by raw read density, such peaks that occur at translation initiation sites, or the periodic stepping of the ribosome (Fig. 3b). In bisulfite sequencing, one might use a mapping function that selectively assigns counts to the genomic positions of C-to-T transitions within a read alignment, enabling CpG islands to be discerned (Fig. 2c). For DMS-seq assays — in which dimethylsulfonate (DMS) alkylates unpaired cytosine and adenine residues in RNA [19] — one would use a mapping function that assigns counts to the alkylated residues, allowing inference of secondary RNA structure (Fig. 2d).

Plastid includes configurable mapping functions applicable to RNA-seq, ribosome profiling, DMS-seq, and a number of other sequencing assays (Table 2). When a novel assay is developed, users can readily implement a mapping function tailored to the experiment. Plastid can then use the new mapping function as a plug-in, enabling immediate application of extant tools to the novel assay. Examples and instructions for writing mapping functions are included in the mapping rules tutorial at https://plastid.readthedocs.io.

**Table 2:** Plastid includes configurable mapping functions that cover many uses cases in sequencing analysis

Method	Map reads	Sample use
Fiveprime	At a fixed offset from their 5′ ends	Ribosome profiling with RNase I (e.g. yeast, human), RNA-seq
Threeprime	At a fixed offset from their 3′ ends	Ribosome profiling with RNase I, RNA-seq
Fiveprime, variable	At an offset from 5′ end determined by read length	Ribosome profiling with RNase I, RNA-seq
Fiveprime, variable and stratified by read length	At an offset from 5′ end determined by read length, partitioning reads of each length into separate arrays	ORF annotation from ribosome profiling data
Center-weighted	Fractionally over entire length, optionally trimming a fixed number of nucleotides from the 5′ and 3′ ends	Ribosome profiling with MNase (e.g. *E. coli* & *D. melanogaster*), RNA-seq

### Encapsulation of discontinuous genomic features

A substantial shortcoming of many existing genomics toolkits is that discontinuous features, such as spliced transcripts, are represented as lists of independently behaving, continuous fragments. For many tasks, this design requires users to perform laborious and error-prone transformations to convert coordinates from the *N* ^*th*^ position of a transcript, to the *I* ^*th*^ position of the transcript’s *J* ^*th*^ exon, and eventually, to the *X* ^*th*^ position in the corresponding genome. Alternatively, users can sacrifice positional information and align their sequencing data to a continuous transcriptome, in this case presuming a priori knowledge of which transcript isoforms are present.

A central difference between Plastid and other toolkits is that Plastid’s encapsulates transcripts and other discontinuous genomic features within single objects, called SegmentChains, that are aware of their own discontinuity (Fig. 3). This design obviates the need to separately track the potentially many exons that together constitute a transcript, and facilitates analysis of phenomena that are easily described in the context of a transcript, but discontinuous in the genome, such as a translational pause site in ribosome profiling data. Thus, users can take advantage of the additional information preserved by aligning reads to a genome, while retaining the convenience of aligning to a transcriptome.

SegmentChains are also useful for analyses that simultaneously consider transcript isoforms that share genomic coordinates, such those implemented in ORF-RATER [10], a tool we have developed to identify and determine translation rates of potentially overlapping open reading frames from ribosome profiling data. For analyses specifically devoted to transcripts, a subclass of SegmentChain, called *Transcript*, is provided. SegmentChains and Transcripts provide tools for many common operations, including:

- mapping coordinates between various transcript isoforms and the genome (Fig. 3a)
- fetching spliced arrays of genomic sequence, read alignments, or count data at any or each nucleotide position in the SegmentChain or Transcript (Fig. 3b)
- fetching sub-regions of the chain, preserving their discontinuity (Fig. 3c)
- masking sub-regions of the chain, such as repetitive regions, from analysis (Fig. 3d)
- testing for equality, overlap, containment, or coverage of other SegmentChains
- accessing and storing descriptive data, like gene names or IDs, GO terms, database cross references, or notes
- exporting to BED, GTF2, or GFF3 formats, for use with other software packages or within a genome browser

### Simplified access to genomic data

In genomics, there are primarily four categories of data — sequence data, feature annotations (e.g. transcript models, coding regions, origins of replication), quantitative values associated with genomic positions (such as conservation scores), and read alignments — yet numerous file formats have been developed to represent each of these data types. Furthermore, many existing packages treat data of a given type in a manner that depends upon the type of file in which it is stored. Becoming familiar with the diverse idiosyncrasies of these file types — for example, whether transcripts are represented one-exon-per-line and must subsequently linked by probing their IDs (GTF2, GFF3 files) or are captured wholly within single lines (BED, BigBed, PSL) — can be time-consuming and a significant impediment to research.

Plastid provides a minimal set of consistently behaved object types for each category of data, and readers for commonly used file formats in each category (Table 1), allowing investigators to focus on their data rather than its representation on disk (Fig. 1). In particular, Plastid provides readers that parse feature annotations in BED, extended BED, BigBed, GTF2, GFF3 and PSL formats into SegmentChains or Transcripts, optionally reconstructing transcripts from their components in GTF2 or GFF3 formats; quantitative data in bedGraph, wiggle, or BigWig formats into GenomeArrays; and read alignments in BAM or Bowtie’s legacy format into GenomeArrays, using mapping functions to transform the data. Because a number of excellent packages already exist for parsing nucleotide sequence, Plastid does not implement new readers for sequence data. However, its tools are compatible with any sequence reader that returns dictionary-like objects, such as those in Biopython (for data in FASTA, GenBank, EMBL, and many other formats; [20]) and twobitreader (for 2bit files; [21]).

### Command-line scripts

In addition to the library it provides for EDA, Plastid includes a number of command-line scripts that implement sequencing workflows commonly used in genomics and NGS analysis (Table 3). While similar implementations exist in other toolkits, Plastid’s scripts are distinct in their use of mapping functions, which allows them to generalize to many types of data and metrics. For example, Plastid’s *make_wiggle* script generates genome browser tracks from sequencing alignments, and, depending upon the mapping function in use, could export a track of ribosomal P-sites, modified nucleotides, unstructured regions of RNA, 5′ ends of read alignments, or whatever type of biology is accessed by the mapping function.

**Table 3:** Plastid’s command-line scripts automate common analysis tasks

Analysis of count and alignment data	
*counts_in_region*	Count the number of read alignments covering arbitrary regions of interest in the genome, and calculate read densities (in reads per nucleotide and in RPKM) over these regions
*cs*	Count the number of read alignments and calculate read densities (in RPKM) specifically for genes and sub-regions (5′ UTR, CDS, 3′ UTR), correcting gene and sub-region boundaries for overlapping genes
*get_count_vectors*	Fetch vectors of counts at each nucleotide position in one or more regions of interest, saving each vector as its own line-delimited text file
*make_wiggle*	Create wiggle or bedGraph files from alignment files after applying a read mapping rule (e.g. to map ribosome-protected footprints at their P-sites), for visualization in a genome browser
*metagene*	Compute a metagene profile of read alignments, counts, or quantitative data over one or more regions of interest
*phase_by_size*	Estimate sub-codon phasing in ribosome profiling data
*psite*	Estimate position of ribosomal P-site within ribosome profiling read alignments as a function of read length
Manipulation of genomic features	
*crossmap*	Empirically annotate multimapping regions of a genome, given alignment criteria
*gff_parent_types*	Determine parent-child relationships of features in a GFF3 file
*reformat*_*transcripts*	Convert transcripts between BED, BigBed, GTF2, GFF3, and PSL formats
*findjuncs*	Find all unique splice junctions in one or more transcript annotations, and optionally export these in Tophat’s.juncs format
*slidejuncs*	Compare a set of splice junctions to a reference set, and, if possible with equal sequence support, slide discovered junctions to compatible known junctions

In addition, Plastid introduces algorithms and scripts for a number of tasks that are not implemented or are handled substantially differently elsewhere. We highlight a few of these below:

### Maximal spanning windows

**Fig. 5:**
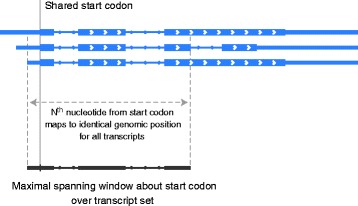
Maximal spanning windows enable isoform-independent analysis. A maximal spanning window over a set of transcripts (or other genomic features) is defined as the largest possible window surrounding a shared landmark (in this example, a start codon; vertical line), over which the *N* ^th^ nucleotide from the landmark in each transcript corresponds to the same genomic position. Maximal spanning windows thus enable position-wise analysis over fractions of genes when isoform distributions are unknown. Plastid uses maximal spanning windows for metagene analysis, measuring sub-codon phasing in ribosome profiling, and estimating ribosomal P-site offsets

Many nucleotide-resolution analyses require prior knowledge of which transcript isoforms are present, but such knowledge is frequently unavailable. For this circumstance, Plastid introduces the use of *maximal spanning windows* (Fig. 5) as an approach to isoform-independent analysis. Briefly, a maximal spanning window is defined as a span of nucleotides surrounding a landmark (e.g. a start codon), in which each position relative to the landmark maps to the same genomic coordinate across every member of a group of transcripts (or other features). Thus, a gene’s maximal spanning window captures the range of feature positions whose distances to each other and to a landmark are independent of whatever transcript isoform(s) that may be expressed.

The use of maximal spanning windows provides a number of advantages over other strategies when isoform distributions are uncertain. A commonly used alternative strategy is to choose a single, “canonical” transcript isoform from each gene to include in analysis. This approximation is appropriate in some circumstances, but is variably inaccurate when comparing across cell lines or culture conditions. Another strategy is to treat all transcript isoforms as independent entities. But, in the absence of corrections downstream, this practice can yield double-counting of read alignments and regions when multiple isoforms overlap. Restricting analysis to each gene’s maximal spanning window minimizes the problems inherent in both of these strategies insofar the quality of a given genome annotation allows.

Plastid contains tools that generate a maximal spanning window surrounding a landmark of interest (such as a start codon) for each gene (or, more generally, any user-specified group of features) in a genome annotation. To do so, Plastid makes use of *landmark functions* that identify a landmark of interest, if present, within a single transcript. The landmark function is applied to each of a gene’s transcripts, and, if the genomic positions of their landmarks are identical (e.g. all start codons match the same genomic coordinate, even if at different coordinates within each transcript), then Plastid’s window-generating toolkit bidirectionally examines each position on each transcript at increasing distance from the landmark until corresponding positions on all transcripts no longer map to the same genomic position. If all transcripts from a given gene do not share the same genomic landmark coordinate (contain different start codons), then the maximal spanning window surrounding that landmark is of zero-length, and excluded from analysis.

Plastid includes landmark functions that identify start and stop codons, and includes instructions for writing functions to programmatically identify other landmarks, such as peaks in sequencing data or nucleotide motifs within a region of interest. Plastid can use maximal spanning windows for estimation of gene expression or for metagene analysis (described below) for any type of sequencing data, and, in the case of ribosome profiling, additionally uses maximal spanning windows for estimation of P-site offsets and sub codon phasing.

### Metagene analysis

**Fig. 6:**
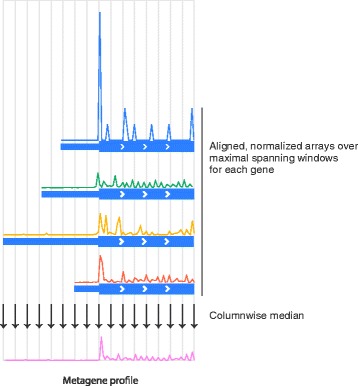
Metagene profiles reveal genomic signals. Schematic of metagene analysis. Normalized arrays of quantitative data (e.g. ribosomal P-sites; top) are taken at each position in the maximal spanning windows of multiple genes. These arrays are aligned at a landmark of interest (here, a start codon), and the median value of each column (nucleotide position), is taken to be the average (bottom)

Noise can obscure important biological signals within individual samples, but such signals frequently appear in population averages. For nucleotide-resolution analysis of NGS data, one particularly useful average is a *metagene profile,* in which arrays of quantitative data, corresponding to each position of a gene or region of interest, are aligned at some landmark — such as a start codon [1], or the beginning of a region encoding a signal peptide [22] — and a position-wise average is taken over the aligned arrays (Fig. 6). Metagene profiles have been used to reveal numerous biological signals, such as peaks of ribosome density at start or stop codons [1], ribosomal pauses over polybasic signals [23], and sites of engagement of hydrophobic nascent chains by the signal recognition particle [22].

Plastid’s metagene toolkit is unique in its use of maximal spanning windows to obtain isoform-independent arrays of data for each individual gene. These arrays are then aligned at the position corresponding to the landmark and a column-wise median is taken at each position. Because users can modify or define both landmark functions and mapping functions, Plastid’s tools can be used to obtain position-wise averages of arbitrary types of data, surrounding virtually any landmark, in arbitrarily grouped sets of regions.

### Multimapping regions of the genome

Specific regions of the genome ― such as transposable elements, pseudogenes, and paralogous coding regions ― can yield sequencing reads that *multimap*, or align equally well to multiple regions of the genome. It is frequently desirable to exclude such regions from analysis, as these introduce ambiguity into sequencing data. However, because a read’s ability to multimap is a function of both its length and the number of mismatches tolerated during alignment, specific experimental regimes require custom annotation of multimapping regions in the genome. Plastid includes a script called *crossmap* that empirically determines which regions of the genome yield multimapping reads of a given length at a permitted number of mismatches.

Elaborating an approach developed in [1], *crossmap* conceptually divides the genome into all possible sequencing reads of length *k*, and then aligns these back to the genome allowing *n* mismatches, where *k* and *n* are given by the user. When a read aligns equally well to multiple regions of the genome under these criteria, its point of origin is flagged as multimapping. *crossmap* exports all multimapping regions as a BED file, which can be subsequently used to mask such regions of the genome from analysis in any of Plastid’s command-line scripts or interactive tools.

## Results and discussion

### Manipulation of data at nucleotide resolution

In its earliest days, next-generation sequencing was used principally for reconstruction of genomes, and, with the advent of RNA-seq, for estimation of gene expression levels. In the first case, the sequences of reads captured the relevant biology, and in the second, the scalar number of read alignments covering an exon or transcript satisfied most experimental needs.

At present, many NGS assays explore biological questions with nucleotide resolution. These assays have created a need for analytical tools that enable users to manipulate data nucleotide-by-nucleotide robustly and easily. Plastid introduces several data models tailored specifically to this way of working: First, mapping functions convert the relevant properties of read alignments into arrays of decoded information, and thus create an important bridge between NGS assays and the analytical tools offered by the SciPy stack [18]. Second, SegmentChains and Transcripts enable users to manipulate quantitative data and feature annotations with nucleotide precision, in genomic or transcript-centric coordinates. Thus, patterns in data can easily be used to annotate new features, and features can be arbitrarily sub-divided, joined, or exported in standard formats, enabling their use in other pipelines and visualization in genome browsers. Finally, maximal spanning windows offer a novel and rigorous approach to uncertainties created when multiple transcript isoforms might be present, a common circumstance when studying higher eukaryotes.

### Ease of use

One of Plastid’s design goals is to lower the barrier to entry for genomic analysis. To this end, Plastid’s design focuses on simplicity and, when possible, use of biological analogies. Plastid therefore introduces a minimal set of classes, and instead favors existing and commonly-used data structures (such as NumPy arrays) and file formats (e.g. BED and GTF2), whenever possible. Data that cannot be captured in standard formats are formatted as tab-delimited tables, which can readily be manipulated in Python (using Pandas [24]), R, or even Excel.

To facilitate reading, re-reading, or writing code, Plastid’s classes, methods, and functions are modeled upon biological idioms and, when possible, given human-readable names. This design enables users to leverage knowledge of biology when familiarizing themselves with Plastid, and also to write code that, using the concrete language of biology, is more easily interpreted by others.

Finally, to enable users, we have written extensive documentation with tutorials and walkthroughs of various types of analysis, as well as a test dataset tailored to those walkthroughs. These are available at https://plastid.readthedocs.io.

### Extensibility

Plastid is designed to be both modular and easily extended, and includes well-defined and documented APIs. In addition, Plastid includes entrypoints to register new mapping functions and their command-line arguments with Plastid’s command-line scripts, enabling advanced users to share their extensions with others.

Plastid also includes script writing tools for implementing new workflows. These include argument parsers that read data in supported file formats into Plastid’s standard objects, enabling developers, like users, to remain agnostic of file formats. Plastid also includes extensions to Python’s warning control system that give developers more finely-grained control over how to group and limit warnings displays, which can be numerous when operating on large genomics datasets.

## Conclusions

Plastid is a genomics and NGS analysis toolkit that offers unique tools for decoding information from read alignments and manipulating data at nucleotide-resolution. Plastid’s design enables it to retain generality and flexibility across assays while remaining user friendly. Thus, we and others have used Plastid to analyze data from numerous NGS assays, including ribosome profiling, RNA-seq, DMS-seq, and bisulfite sequencing.

Plastid’s utility derives not only from the introduction of mapping functions, SegmentChains, and maximal spanning windows, but also from a design intent that focuses on simplicity, consistency, and integration with other packages: biological data are represented through unified interfaces regardless of the underlying file format; these interfaces are modeled on biological idioms; and, importantly, these interfaces integrate seamlessly with the SciPy stack. Thus, both novice users and experienced bioinformaticians have found Plastid useful. Versions of Plastid have been used in a number of publications [10, 25] and manuscripts in progress (personal communications from C.A. Gross, M. Schuldiner, and N. Bellletier & E.A. Gavis), and is the genomic engine of our ORF annotation software, ORF-RATER [10].

## Availability and requirements

### Source code

Plastid is released under the BSD 3-Clause license. Official releases are available in the Python Package Index at http://pypi.python.org/pypi/plastid. Development versions are available at the project’s home page, https://github.com/joshuagryphon/plastid. Examples, user documentation, and technical information are available at http://plastid.readthedocs.io. The version discussed in this article is Plastid 0.4.6.

### Computing requirements

Plastid is platform-independent and runs on Python 2.7 and Python 3.3 or greater. It depends on Cython [26], numpy [17], and Pysam [27] for compilation, and additionally SciPy [18], matplotlib [28], pandas [24], Biopython [20], twobitreader [21], and termcolor [29] for runtime.

Plastid runs well on laptops, but system requirements scale with the complexity of the genome annotation and the number of read alignments in a dataset. The minimum amount of RAM we recommend for *S. cerevisiae* and other small genomes is 1 GB; for mid-sized genomes like *D. melanogaster,* 4 GB; and 8 GB for vertebrate or plant genomes. Run times and memory usage for worst-case scenarios under a variety of scripts included in Plastid are shown in Table 4.

**Table 4:** Computing requirements for genomes and datasets of varying size

Test	Organism	Run time (hh:mm:ss)	Peak memory usage (MB)
Read counting	Yeast	00:01:18 ± 00:00:01	255 ± 0
Read counting	Fly	00:36:34 ± 00:00:03	1138 ± 7
Read counting	Human	00:19:56 ± 00:00:01	1053 ± 2
Manipulate annotations	Yeast	00:00:27 ± 00:00:02	467 ± 0
Manipulate annotations	Fly	00:03:37 ± 00:00:03	2620 ± 1
Manipulate annotations	Human	00:18:42 ± 00:01:49	4419 ± 1
Export browser track	Yeast	00:00:58 ± 00:00:00	281 ± 1
Export browser track	Fly	00:09:05 ± 00:00:40	2452 ± 7
Export browser track	Human	00:06:11 ± 00:00:03	537 ± 0
Build crossmap	Yeast	00:00:35 ± 00:00:00	100 ± 0
Build crossmap	Fly	00:10:44 ± 00:00:10	328 ± 7
Build crossmap	Human	04:11:51 ± 00:06:32	130 ± 1

Four command-line scripts were executed on yeast, fly, and human datasets. Runtimes and peak memory usage are given as the mean ± standard deviation of three replicates. See methods for details

### External datasets and software used in this study

Sequencing datasets supporting the conclusions of this article are available in the the SRA [30] under accession numbers SRR1562907 (ribosome profiling, [22]); SRR019600-20 and SRR20276-20282 (bisulfite sequencing, [31]); and SRR1057939 (DMS-seq, [19]). Data were visualized in the Integrative Genomics Viewer [32] and modified in Adobe Illustrator CS6. Code syntax was highlighted using Pygments version 2.2 [33].

For Fig. 2, ribosome profiling dataset SRR1562907 [22] was stripped of 3′ cloning adaptors (CTGTAGGCACCATCAAT), and aligned to the yeast reference genome (SGD R64.1.1) using Tophat 2.1.0 [34]. Ribosomal P-sites were assigned to be 15 nucleotides from the 3′ end of 25-35mers. Bisulfite sequencing data were pooled from SRA datasets SRR019600-20 and SRR20276-20282 [31], stripped of 3′ cloning adaptors (AGATCGGAAGAGC) and aligned to the human reference genome (UCSC hg38p3; [35]) using Bismark 0.14.4 [36]. Methylation was determined from Bismark calls by parsing the *XM* flag of each alignment following the specification in [36]. DMS-seq dataset SRR1057939 [19] was downloaded and aligned to human genome sequence (Ensembl GrCh38.78; [37]) using Tophat [34]. Counts were assigned to alkylated residues, estimated to be 1 base 5′ of the read alignment, in the direction of the alignment. SECIS elements and their structure predictions were identified using SeciSearch 2.19 [38].

For Table 4, all tests were run on a single 2.7 GHz Intel Core i7-5700 CPU on an MSI Apache Pro QE2 laptop, in a virtual machine running Ubuntu 14.04 with 10 Gb of RAM, except for *Build crossmap*, which used two cores. Runtimes and memory usage were monitored using Memory Profiler version 0.32 [39]. For tests on yeast, we used the annotation and genome assembly from SGD R64.1.1 [40], 5′ and 3′ UTR definitions from [41] and [42], and ribosome profiling dataset SRR1562907. For tests on the fly genome, we used the annotation and genome assembly from FlyBase r5.54 [43] and merged ribosome profiling datasets from [26] (SRA numbers SRR942868-77). For tests on the human genome, we used all APPRIS-scored [44] transcripts from Ensembl annotation GrCh38.81 [37], the hg38 genome assembly from UCSC [35], and ribosome profiling dataset SRR1976443. All genome annotation files were converted to GTF2 format. Sequence was in FASTA format with the exception of hg38, which was kept as a 2bit file. Alignments of all sequencing reads were kept in BAM format. For tests that used read alignments, alignments were mapped as follows for each organism: 15 nucleotides from the 3′ end of the read for *S. cerevisiae* (modified from [1]), center-weighted mapping for *D. melanogaster* [25], and using a variable offset for *H. sapiens* [2].

For each organism dataset, a series of tests were conducted. In *Manipulate annotations*, all transcripts, genes, exons, and coding regions within a chromosome were compared and modified in multiple ways using Plastid’s *cs* script, executed as *cs generate /tmp/foo --annotation_file gtf_file.gtf --sorted*. In *Read counting*, read counts and densities were tabulated for all transcripts in a genome annotation using the *counts_in_region* script, executed as *counts_in_region /tmp/foo --count_files bam_file.bam--annotation_files* *gtf_file.gtf --sorted* [*--threeprime--offset 15* for yeast | *--fiveprime_variable p_off.txt* for human | *--center --nibble 12* for fly]. In *Build crossmap*, an empirical annotation of which regions in a given genome give rise to multimapping reads was empirically determined by slicing the genome sequence into *k*-mers and counting the number of times each *k*-mer aligned to the genome using Plastid’s *crossmap* script, which internally used Bowtie version 1.1.2 [45]. The *crossmap* script was executed as *crossmap -k 26 --mismatches 0 -p 2 --sequence_file file.*[*fa* | *2bit*] *--sequence_format* [*FASTA* | *2bit*] */path/to/bowtie/index /tmp/foo*.

## Abbreviations

CAGE-seq: Cap-analysis gene expression, for identification of 5′ ends of eukaryotic messenger RNAs
ChIP-seq: Chromatin immunoprecipitation sequencing, for probing sites of DNA::protein interaction
DMS-seq: Dimethylsulfonate sequencing, for probing RNA structure
EDA: Exploratory data analysis
GB: Gigabyte
hh:mm:ss: Time expressed as hours:minutes:seconds
MB: Megabyte
NGS: Next-generation sequencing
UTR: Untranslated region
